# Eco-friendly synthesis of *Balanites aegyptiaca*-derived selenium nanoparticles: extract and assessment of their anticancer, antimicrobial, cytogenetic and molecular docking insights

**DOI:** 10.1038/s41598-026-35358-z

**Published:** 2026-02-02

**Authors:** Mohamed I. M. El-Zaidy, Heba G. Ayoub, Gehan El-Akabawy, Amira A. Ibrahim, Mohamed Abdel-Haleem

**Affiliations:** 1 Department of Chemistry, Faculty of Science, Zagazig University, Zagazig, 44519 Egypt; 2 High Technological Institute for Applied Health Sciences, K 36 Cairo–Ismailia Road, Cairo, Egypt; 3 Department of Basic Medical Sciences, College of Medicine, Ajman University, Ajman, United Arab Emirates; 4 Centre of Medical and Bio-allied Health Sciences Research, Ajman University, Ajman, United Arab Emirates; 5 Department of Anatomy and Embryology, Faculty of Medicine, Menoufia University, Menoufia, Egypt; 6 Botany and Microbiology Department, Faculty of Science, Arish University, Al-Arish, 45511 Egypt; 7 Department of Botany and Microbiology, Faculty of Science, Zagazig University, Zagazig, 44519 Egypt

**Keywords:** Balanites aegyptiaca, SeNPs, Phenolic compounds, Anticancer activity, Antimicrobial activity, Chromosomal aberrations, Biochemistry, Biological techniques, Biotechnology, Cancer, Chemical biology, Chemistry, Drug discovery, Microbiology

## Abstract

This study reports the eco-friendly synthesis of selenium nanoparticles (SeNPs) using the methanolic extract of *Balanites aegyptiaca* mesocarp and evaluates their biological activities. The synthesized spherical SeNPs (average size: 2.82 nm) were characterized by TEM, FESEM, and UV–Vis spectroscopy, confirming that phenolic compounds serve as both reducing and stabilizing agents. HPLC analysis revealed eight major phenolics, with gallic acid, chlorogenic acid, and daidzein being the predominant compounds. The SeNPs exhibited strong cytotoxicity against HCT-116 colorectal cancer cells (IC₅₀ = 30.03 µg/mL), potent antibacterial activity against *Klebsiella pneumoniae*,* Escherichia coli*, and *Enterococcus faecalis*, and induced concentration-dependent cytogenetic effects in *Vicia faba* root tips. Molecular docking studies suggested that phenolic compounds effectively interact with the CDK4 active site, supporting their potential anticancer properties. These findings highlight *B. aegyptiaca*-derived SeNPs as promising candidates for biomedical applications.

**Supplementary Information:**

The online version contains supplementary material available at 10.1038/s41598-026-35358-z.

## Introduction

Nanomaterials have garnered extensive attention due to their unique thermal, optical, electrical, and physical properties. Among these, semiconductor nanoparticles are particularly important owing to their exceptional nonlinear properties, saturable absorption, and optical biostability, which distinguish them from other types of nanomaterials^1^. Selenium (Se), an essential trace element, has recently gained significant interest due to its critical physiological role in human health^2,3^. It participates in the metabolism of thyroid hormones and immune function, and its incorporation into antioxidant enzymes protects against free radical–induced cellular damage. Selenium deficiency has been linked to a wide range of diseases, including cancer, cardiovascular disorders, and inflammatory conditions^4^. However, excessive or prolonged selenium intake may induce toxic effects^5^.

In recent years, the biological synthesis of nanoparticles using microbes and plant extracts has emerged as a promising alternative to physical and chemical methods. Plant-based synthesis offers several advantages, including simplicity, safety, low cost, and sustainability, as it eliminates the need for maintaining microbial cultures^6^. In these green synthesis approaches, plant metabolites act as both reducing and stabilizing agents. Various biomolecules found in plant extracts, such as sugars, amino acids, enzymes, tannins, polysaccharides, phenolic compounds, flavonoids, saponins, and proteins, play crucial roles in reducing selenium ions and stabilizing the resulting nanoparticles^7^. Because nanoparticles tend to aggregate, a monolayer of polymer or surfactant is often used to control growth and minimize inter-particle interactions.

Several plants have been successfully employed for the biosynthesis of SeNPs, including orange peel, *Emblica officinalis*,* Moringa oleifera*,* Aloe vera*,* Allium sativum*,* Cassia auriculata*,* Bougainvillea spectabilis*,* Asteriscus graveolens*,* and Vitis vinifera*^8–11^. Many of these plants are well known for their diverse biological activities. *Emblica officinalis and Cassia auriculata* exhibit antioxidant and antileukemic properties, respectively. Biosynthesized SeNPs have demonstrated potent anticancer effects against HepG2 cells^12–17^. Extracts from Hawthorn plants show chemotherapeutic potential against human liver cancer^18^, while bioactive compounds from *Zingiber* display antimicrobial activity against *Proteus* species^19^. Similarly, Ashwagandha has been reported to exhibit antibacterial activity against *Klebsiella pneumoniae* and *Bacillus subtilis*^20^. Fenugreek has demonstrated inhibitory effects on breast cancer (MCF-7) cell growth^21^, while Java tea showed cytotoxic effects on L6 cell lines^22^. *Lavender leucas* exhibited antibacterial and antifungal properties^23,24^. Additionally, garlic demonstrated antimicrobial activity^25^, and *Clausena dentata* showed insecticidal potential against mosquito vectors^26^.

Colorectal cancer represents one of the leading causes of cancer-related morbidity and mortality worldwide^27^. The HCT-116 human colon adenocarcinoma cell line is commonly used as an in-vitro model for studying colorectal carcinoma because of its strong proliferative capacity, invasive potential, and partial resistance to conventional chemotherapeutic agents^28^. These biological characteristics make HCT-116 cells an excellent model for evaluating novel anticancer strategies, including the therapeutic efficacy of selenium-based nanomaterials^29^.

The plant mediated production of SeNPs has also emerged as an effective strategy to combat microbial infections^30^. Compared to conventional antimicrobial agents, biogenic SeNPs demonstrate enhanced stability, broad-spectrum activity, and environmentally benign synthesis routes^31^. Their nanoscale dimensions and distinctive surface chemistry facilitate strong interactions with bacterial cell walls and intracellular targets, suggesting their potential as alternative antimicrobial agents^32^. Gram-negative bacteria such as *K. pneumoniae* ESA254 and *E. coli* ESA253, and Gram-positive strains including *Enterococcus faecium* ESA22, have shown high susceptibility to SeNPs^33^. These findings reinforce the potential of plant-derived SeNPs as novel antibacterial candidates against multidrug-resistant pathogens.

With the increasing use of nanomaterials in various industrial and biomedical sectors, including pharmaceuticals, cosmetics, food production, paints, and electronics, daily human and environmental exposure have significantly increased^34^. However, their physicochemical properties and potential toxicity remain inadequately understood. Consequently, evaluating the genotoxic and cytological impacts of nanomaterials is critical, particularly in plants used as biological indicators such as *A. cepa*,* Lens culinaris*,* and Vicia faba*^35,36^. Nanoparticles may induce chromosomal abnormalities, cell degeneration, and the generation of reactive oxygen species (ROS), leading to DNA damage and mitotic disturbances^37,38^. Observed cytogenetic effects include chromosome breakage, lagging, spindle dysfunction, stickiness, fragmentation, gaps, and polyploidy^39,40^.

Furthermore, molecular docking has become a crucial computational tool for predicting interactions between nanoparticles or bioactive molecules and their protein targets. It provides valuable insights into binding sites, affinities, and mechanisms of action. This approach is particularly useful for exploring antimicrobial and anticancer mechanisms by identifying how SeNPs or plant derived compounds interact with key enzymes or receptors^41^.

This work has the potential to fill critical gaps in the current state of biomedical and nanotechnology research, making it a significant contribution. There is an immediate need for novel, secure, and economically viable treatment options due to the worrisome growth of multidrug-resistant infections and the rising worldwide burden of diseases such as colorectal carcinoma. The bioactivity, low toxicity, and environmentally benign manufacturing of plant-mediated SeNPs make them promising candidates for use in biomedicine. This research demonstrates the effectiveness of SeNPs against therapeutically relevant bacteria, offering mechanistic insights into their mode of action through a combination of experimental antimicrobial studies and molecular docking analysis. To further assure the biosafety of SeNPs for prospective therapeutic uses and to evaluate their genotoxic potential, chromosomal aberration screening is crucial. Ultimately, these results contribute to the development of new nanomedicine approaches, which may lead to future treatments for cancer and infectious diseases.

## Materials and methods

### Plant collection

2 kg of *B. aegyptiaca* fruits from sellers in the Aswan district. Dr. Alaa Eldin Sayed Ewase of Egypt’s Ministry of Environment conducted the identification and authentication of the specimens. Ewase works for the Biodiversity Administration in Cairo. In the herbarium of the National Research Center (NRC) in Giza, Egypt, with the global code (CAIRC), there is a voucher specimen with the number 0034. A yield of 200 g was achieved by isolating the mesocarp of *B. aegyptiaca*, shading it at room temperature, and then washing the fruits many times with distilled water. A fine powder was produced by grinding the mesocarp components. The powder was stored at 4 °C until it was time for examination.

### Extraction

The dried *B. aegyptiaca* mesocarp powder (200 g) underwent defatting using petroleum ether (60–80 °C), followed by n-hexane, and finally methanol, utilizing a Soxhlet apparatus. The crude extracts were concentrated to dryness using a Rotavapor (Heidolph, Germany). The extracts, weighing 18.41 gm, 14.31 gm, and 11.22 gm, respectively, were stored at -4 °C for subsequent analyses.

### Chemicals reagent

All standards, including gallic acid, chlorogenic acid, catechin, caffeic acid, syringic acid, ferulic acid, naringenin, and daidzein, utilized for identification and quantification, were acquired from Sigma-Aldrich. The solvents employed were sourced from Merck (Germany).

### High performance liquid chromatography

In Giza, Egypt, the National Research Center (NRC) carried out the HPLC analysis. For this analysis, the methanolic extract of *B. aegyptiaca* mesocarp was run through an Agilent 1260 HPLC system (Agilent Technologies, Santa Clara, CA, USA) that had a quaternary pump (G1311C), UV-Vis detector (G1314B), and autosampler (G1329B). A polyphenolic chemical reference solution was prepared at a concentration of 1 milligram per milliliter before injection into the HPLC apparatus. Elution was performed using Solvent B, which consisted of methanol and a linear gradient of 0.05% phosphoric acid in water (pH 2.5), flowing at a rate of 1 mL/min. In the subsequent steps, the different time intervals for the gradient program were set at 0%, 5%, 8%, 40%, 50%, 75%, 20%, 22%, 90%, and 100% (B-100%), respectively. The chromatograms were captured for 25 min at 260 nm. For the linearity calculations, a concentration range of 0.1 µg/ml to 10 εg/ml was utilized, and a resolution of 1.5 was maintained between the peaks^42^.

### Biosynthesis of senps

The green synthesis of SeNPs was carried out according to the method of Ibrahim et al.^43^ with slight modifications. The synthesis was performed by adding 100 ml of diluted methanol extract (4 mg stock extract/100 ml distilled water) of *B. aegyptiaca* fruit to 900 ml of freshly prepared aqueous sodium selenite (Na_2_SeO_3_) (10 mM) at 60 °C for 12 h on a hot plate with a magnetic stirrer (1000 rpm). The SeNPs were isolated and purified by centrifugation at 8000 rpm for 20 min, followed by redispersion of the nanoparticle pellet in absolute ethanol. The pure nanoparticles were kept at 4 °C for analysis after air drying.

### Characterization of senps

The SeNPs were initially analyzed using a digital pH meter (Eutech Cyberscan pH 300). Then, the Rigol ultra-3660 UV-vis spectroscopy was used within the range of 200–800 nm. Subsequently, FESEM was used to examine the morphology, scale and surface area of the SeNPs. The SeNPs solutions were ultrasonicated for 15 min at room temperature, and one drop of the sample was put on a glass slide. After drying, the glass slide was coated with gold and analyzed under a Field Emission Scanning Electron Microscope (Zeiss EvoMA 10, Germany). Se nano powder was suspended in ethanol, sonicated, then coated onto a copper grid and allowed to dry and examined by JEOL-2100 HRTEM and FTIR (IRPrestige-21^®^, Germany) in a range of 500 to 3500 cm-1 using the potassium bromide pellet method, where the formula was lyophilized and mixed with potassium bromide (1:10) and applied to the instrument. The prepared formula’s average particle size and zeta potential were measured by dynamic light scattering analysis (Zeta sizer Nano ZS, Malvern In­struments, Malvern, UK)^44,45^.

### Cytotoxicity assay

This study’s cancer cell line was procured from Sera and the Institute of Vaccines. Due to the need for precise control, all studies involving these cells were conducted in a laboratory setting. The cells were placed in 96-well tissue culture plates with their specialized growth medium and left to develop exponentially for 24 h. The cell density was 5 × 10⁴ cells per well. After that, the test substance was introduced at various concentrations and incubated for an additional 24 h. After that, new growth media were added to each well in a 1:10 ratio with MTT solution (5 mg/mL in PBS). To facilitate formazan production, the cells were incubated at 37 °C for an additional 4 h. To stop the reaction, 100 µL of DMSO was added to dissolve the formazan crystals, and the media containing MTT was removed. To determine if the cells were viable, the microplate reader was used to measure the absorbance at 590 nm. To create survival curves for the treated tumor cells, we first calculated the IC50 (half-maximal inhibitory concentration) for each cell line, which allowed us to ascertain the cytotoxicity of the chemical^46^.

### Molecular docking simulation

Molecular docking studies were carried out using (MOE, version 2014.13) suite to propose and explain the recommended mechanism of action for the bioactive phenolic compounds isolated from *B. aegyptiaca* mesocarp methanol extract as promising (CDK4) protein (PDB: 2W96) inhibitors. Moreover, the co-crystallized glycerol (GOL) inhibitor was inserted into the examined database as a reference standard.

#### Validation of the MOE Docking process

**Fig. 1:**
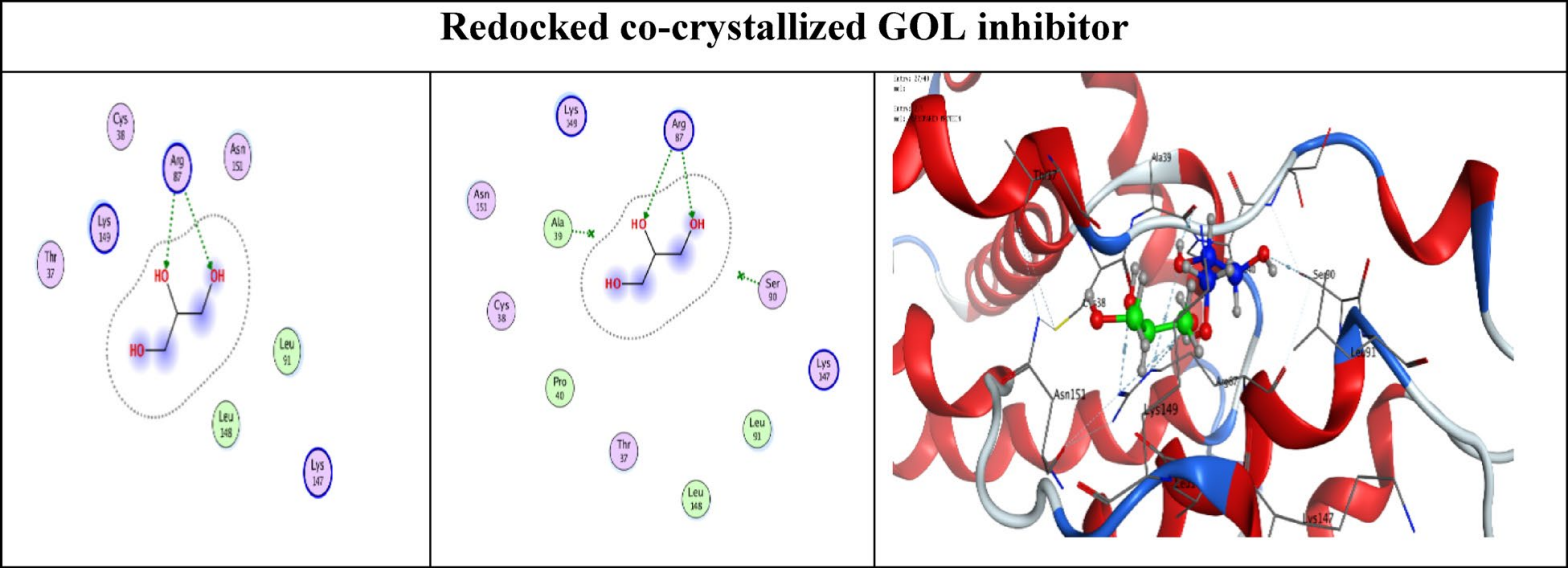
2D and 3D representations show the superimposition between the co-crystallized GOL inhibitor (blue) and the redocked one (green).

First, we redocked the co-crystallized GOL inhibitor inside its binding pocket within the CDK4 target protein. This was done to validate the docking program results for the examined compounds by observing both the binding mode and RMSD value of the redocked GOL inhibitor^47^. Notably, a very close similarity between the redocked (green) and native (blue) poses of the GOL inhibitor, with an RMSD value of 0.6332, indicates a highly accurate performance for the MOE docking program (Fig. 1).

### Preparation of the bioactive phenolic compounds and GOL

The structure of the fifteen bioactive phenolic compounds isolated from *B. aegyptiaca* mesocarp methanol extract was sketched using ChemDraw Professional 15.0. It was introduced into the MOE window and prepared individually for docking according to the steps. Then, all the prepared fifteen compounds, together with the extracted GOL inhibitor, were inserted into a single database to be uploaded for general docking processing.

#### Preparation of the target CDK4 protein receptor

The X-ray structure of the target CDK4 protein receptor was obtained from the Protein Data Bank (PDB) website (ID: 2W96). The target receptor was subjected to the previously mentioned steps in detail^47^.

#### Docking of the database to the CDK4 binding pocket

The previously prepared database was uploaded to the ligand’s site in a general docking process. The docking process specifications were applied as described earlier^48^. The best pose for each studied compound was selected based on the score, binding mode compared to the co-crystallized inhibitor, and RMSD value as well^49^.

### Antibacterial activity of *B. aegypticaca* and senps nanoparticles

One Gram-positive bacterium, *E. faecium* ESA22, and two Gram-negative bacteria, *K. pneumoniae* ESA254 and *E. coli* ESA253, both associated with urinary tract infections, were tested for their antibacterial activity against the *B. aegyptiaca* plant extract and for the green synthesis of SeNPs using this plant extract. Following the guidelines established by the Clinical and Laboratory Standards Institute (2022), this evaluation was conducted in vitro using agar diffusion methods with varying doses of the plant extract and SeNPs. To achieve nanoparticle concentrations of 50, 100, and 150 µg/mL, the *B. aegyptiaca* extract and SeNPs were prepared in 0.5% dimethyl sulfoxide (DMSO, Sigma-Aldrich, USA). The bacterial strains were cultured in LB broth medium at 37 °C for 2 days. Approximately 105 CFU/mL of bacterial pathogens, in 20 µL of LB medium, were added to plates that had been prepared earlier. Once the sterile filter paper discs, with a diameter of 5 mm, had settled, they were immersed in varying concentrations of nanoparticles (50, 100, and 150 µg/mL) along with conventional antibiotics (5 µg/mL), such as Augmentin and Gentianin. The plates were incubated at 37 °C for 24 h after each treatment, and the experiment was repeated three times. Following the incubation period, a ruler was used to measure the diameter of the clear zone, in millimeters (mm), encircling the agar wells. A set of microdilutions was used to find the MIC, or minimal inhibitory concentration.

### Genotoxicity assessment of *B. aegyptiaca* senps using the *V. faba* assay

To evaluate SeNPs made using *B. aegyptiaca* extract, the *V. faba* assay was performed according to a modified version of Grant’s procedure (Grant, 1982). To evaluate the genotoxic, cytotoxic, and phytotoxic effects of SeNPs, root tips of *V. faba* were exposed to different doses. To conduct a comparison study, negative control was included using distilled water. We recorded the percentage of germination and root development after treatment. Next, the meristematic root tips were obtained and preserved. Carnoy’s solution (3:1 ethanol: acetic acid, v/v) was used for fixing, and hydrolysis was carried out in 1 N HCl. Schiff’s reagent was used for staining, and acetic carmine was used for contrast enhancement. Compressing meristematic areas and mounting them in synthetic resin allowed for the creation of slides for cytogenetic examination. The mitotic activity and nuclear damage of 5,000 cells were examined in great detail under a light microscope set at 1000x magnification in this study. The genotoxic potential was evaluated by reviewing nuclear and chromosomal abnormalities at various stages of mitosis, including C-metaphases, multipolar spindles, lagging or fragmented chromosomes, and anaphase and telophase bridges. By dividing the number of cells with abnormalities by the total number of cells tested, we derived the chromosomal aberration index (CA). The micronucleus test was used to evaluate mutagenic effects; the micronucleus index (MN) is the ratio of cells containing micronuclei to the total number of cells. To assess the impact of SeNPs on cell proliferation, the mitotic index (MI) was utilized. We used one-way ANOVA and Dunnett’s test for our statistical analyses, with a significance threshold of 0.05^50^.

### Statistical analysis

Statistical analysis was performed using SPSS software (version 23; IBM SPSS, USA). The software was used for data organization, descriptive statistical analysis, and graphical representation. All results are expressed as mean ± standard error of the mean (SEM). No additional inferential statistical tests were applied. This descriptive statistical approach was used to summarize the experimental data and illustrate overall trends.

## Results and discussion

### Investigation of phenolic compounds of *B. aegyptiaca* fruit using HPLC

**Fig. 2:**
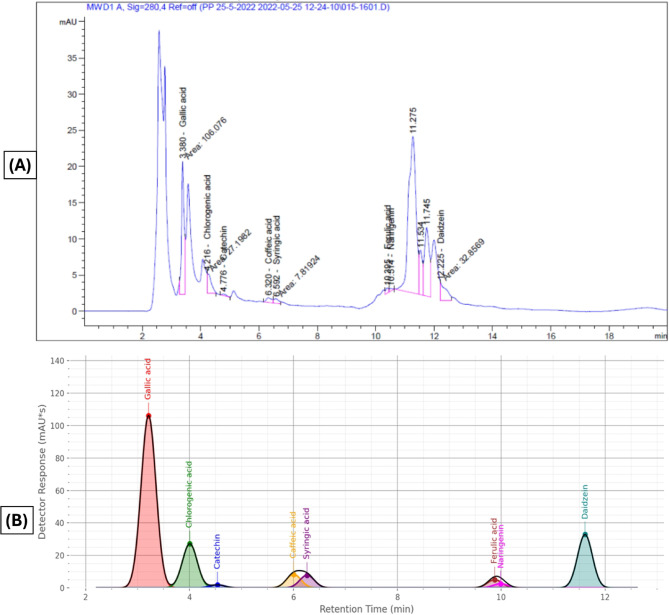
Investigation of the methanolic extract of *B. aegyptiaca* mesocarp using HPLC analysis. (**A**) Representative HPLC chromatogram of the methanolic extract, showing the separation and relative abundance of detected compounds. (**B**) Simulated chromatogram used to predict peak identities and compare with experimental results. Peaks are labelled according to retention times, facilitating the identification of major phenolic constituents in the extract.

The phenolic compounds present in the methanolic mesocarp extract of *Balanites aegyptiaca* were analyzed using HPLC, and the results are presented in Fig. 2; Table 1. Eight phenolic compounds were identified by comparison with standard samples. Gallic acid showed the highest concentration (630.78 µg/g), followed by chlorogenic acid (262.53 µg/g) and daidzein (150.50 µg/g). In contrast, syringic acid (58.33 µg/g), caffeic acid (52.45 µg/g), catechin (34.47 µg/g), ferulic acid (28.56 µg/g), and naringenin (22.49 µg/g) were detected at lower concentrations. These phenolic constituents may contribute to the potential therapeutic properties of the extract.

**Fig. 3:**
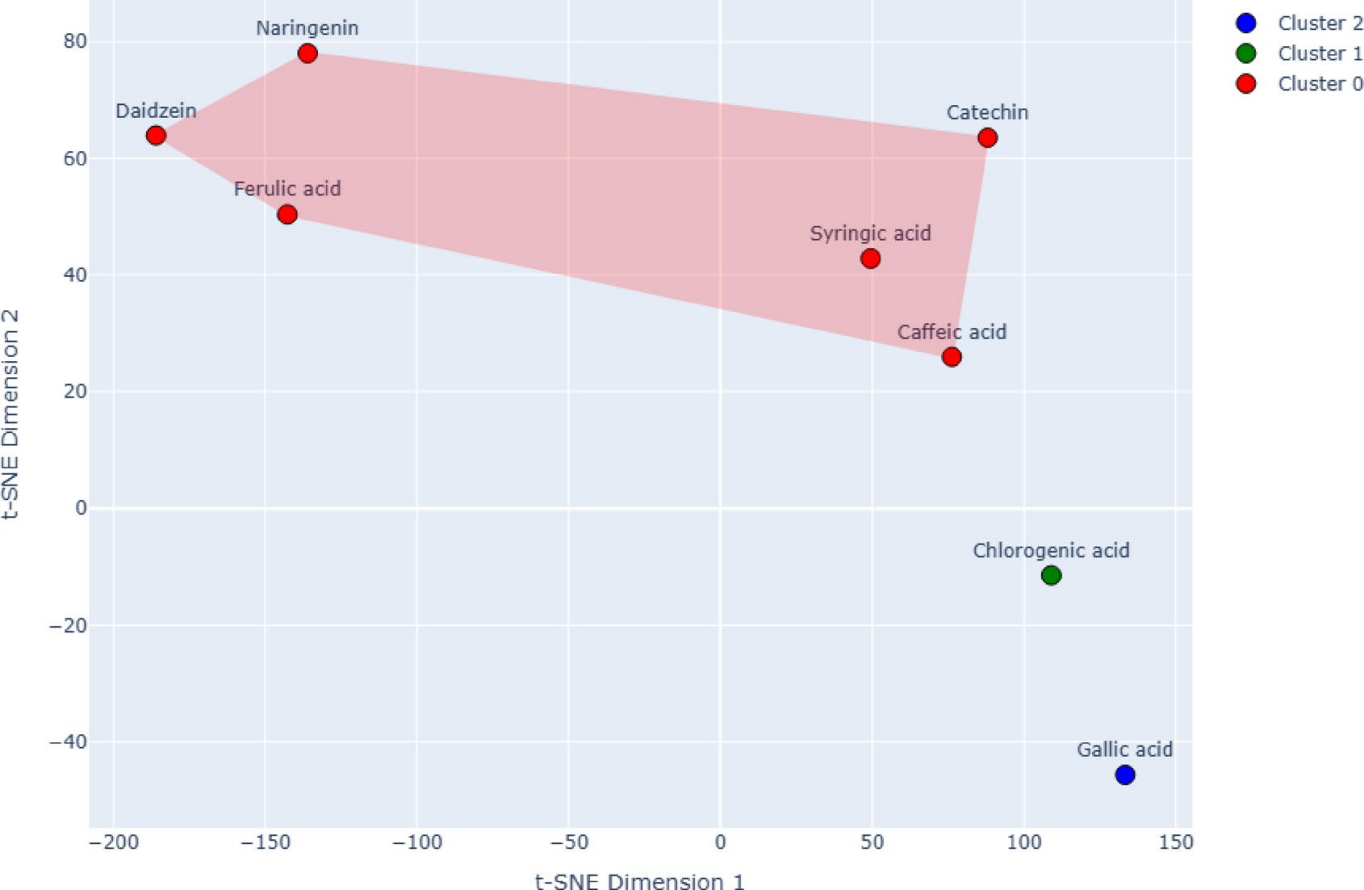
t-SNE Visualization of Phenolic Compounds in *B. aegyptiaca* with KMeans Clustering and Convex Hulls.

The phenolic compounds in the *B. aegyptiaca* fruit extract were successfully separated using multivariate analysis, including principal component analysis (PCA), t-SNE visualization, and K-means clustering. Considering the concentration of the chemicals, peak area, retention time, and clustering, their basic similarities and differences are evident. This method offers a robust and aesthetically appealing approach to analyzing compositional patterns in complex phytochemical datasets, which can guide future research on bioactive component classification and their potential biological consequences (Fig. 3).

**Fig. 4:**
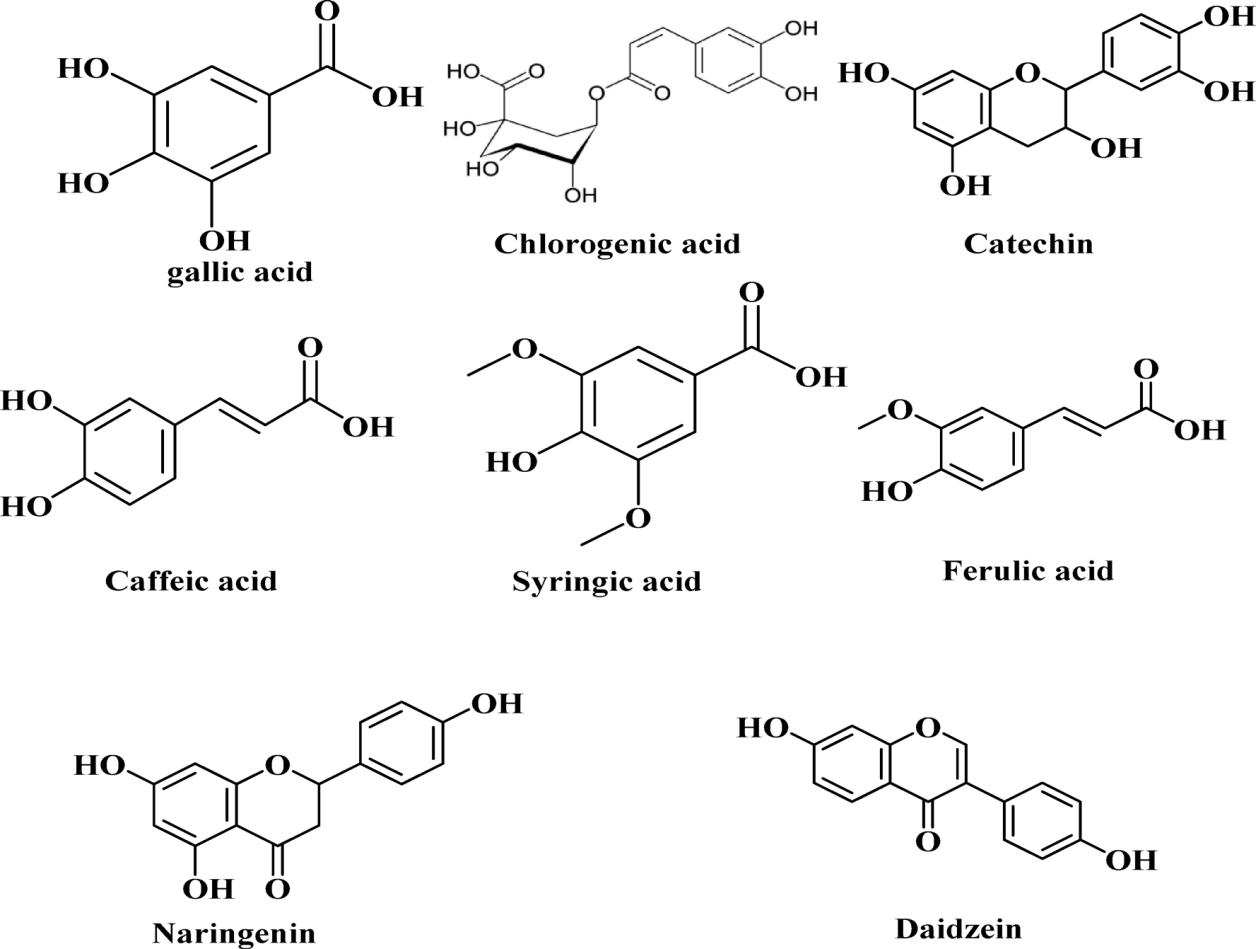
Chemical structures of bioactive phenolic compounds identified in the methanolic extract of *B. aegyptiaca* mesocarp. Each structure corresponds to a compound detected and characterized through HPLC analysis, highlighting the diversity of phenolic constituents present in the extract.

**Fig. 5:**
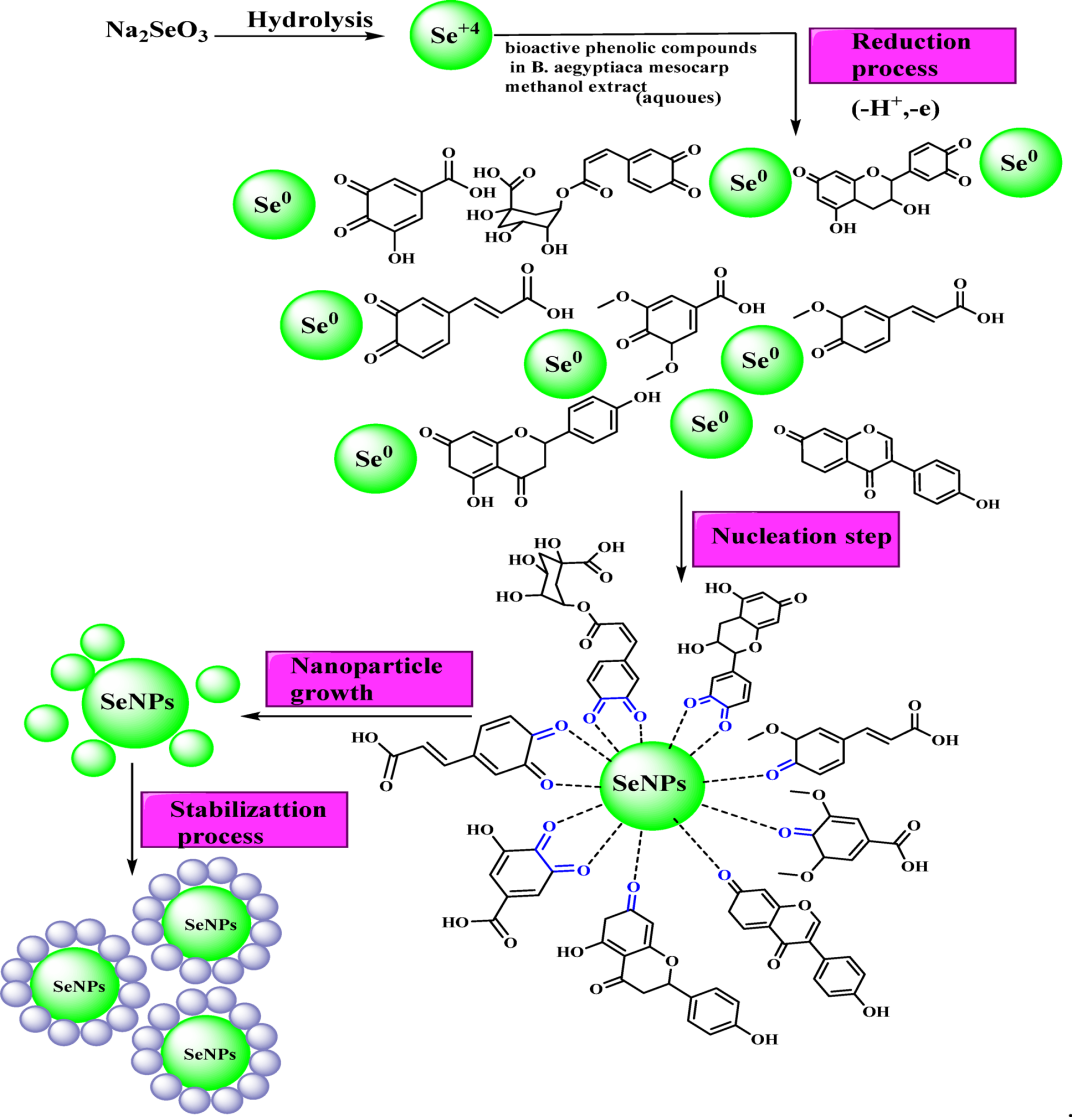
Proposed mechanism for the synthesis of SeNPs using bioactive phenolic compounds from *B. aegyptiaca* mesocarp methanolic extract. The process involves: (i) hydrolysis of Na₂SeO₃ to produce Se⁴⁺ ions, (ii) reduction of Se⁴⁺ to elemental Se⁰ by phenolic compounds, (iii) nucleation leading to initial SeNP formation, (iv) nanoparticle growth, and (v) stabilization of SeNPs through capping by phenolic molecules. Green spheres represent Se species, and arrows indicate the stepwise transformation during the biosynthesis process.

The significant quantity of bioactive phenolic compounds found in *B. aegyptiaca* mesocarp extract (Fig. 4), which may supply electrons for the reduction of Se⁴⁺ ions to SeNPs, is what enables the green synthesis of SeNPs. As seen in (Fig. 5), the carboxyl and hydroxyl groups create a protective layer on the SeNPs’ surface during the procedure. The particles can be stabilized by the steric barrier that this shielding layer can provide^51,52^.

**Table 1:** Bioactive phenolic compounds identified of *B. aegyptiaca* mesocarp methanol extract using HPLC chromatogram.

Peak No.	*R*_t (min)_	Compounds	Area[mAU*s]	Conc. (µg/ml(	Conc. (µg/g)
1	3.380	Gallic acid	106.08	8.52	630.78
2	4.216	Chlorogenic acid	27.20	3.54	262.53
3	4.776	Catechin	1.92	0.47	34.47
4	6.320	Caffeic acid	8.35	0.71	52.45
5	6.592	Syringic acid	7.82	0.79	58.33
6	10.395	Ferulic acid	5.10	0.39	28.56
7	10.514	Naringenin	2.64	0.30	22.49
8	12.225	Daidzein	32.86	2.03	150.50

### Biosynthesis of selenium nanoparticles

The biosynthesis of selenium nanoparticles using *Balanites aegyptiaca* extract is proposed based on the obtained experimental results. Phytochemical constituents present in the plant extract, such as phenolic compounds, are believed to act as reducing and stabilizing agents during the conversion of sodium selenite into selenium nanoparticles. The formation and stabilization of the nanoparticles were supported by spectroscopic analyses, indicating the involvement of functional groups present in the extract.

### Characterization of green synthesized senps

#### Change in visual color

**Fig. 6:**
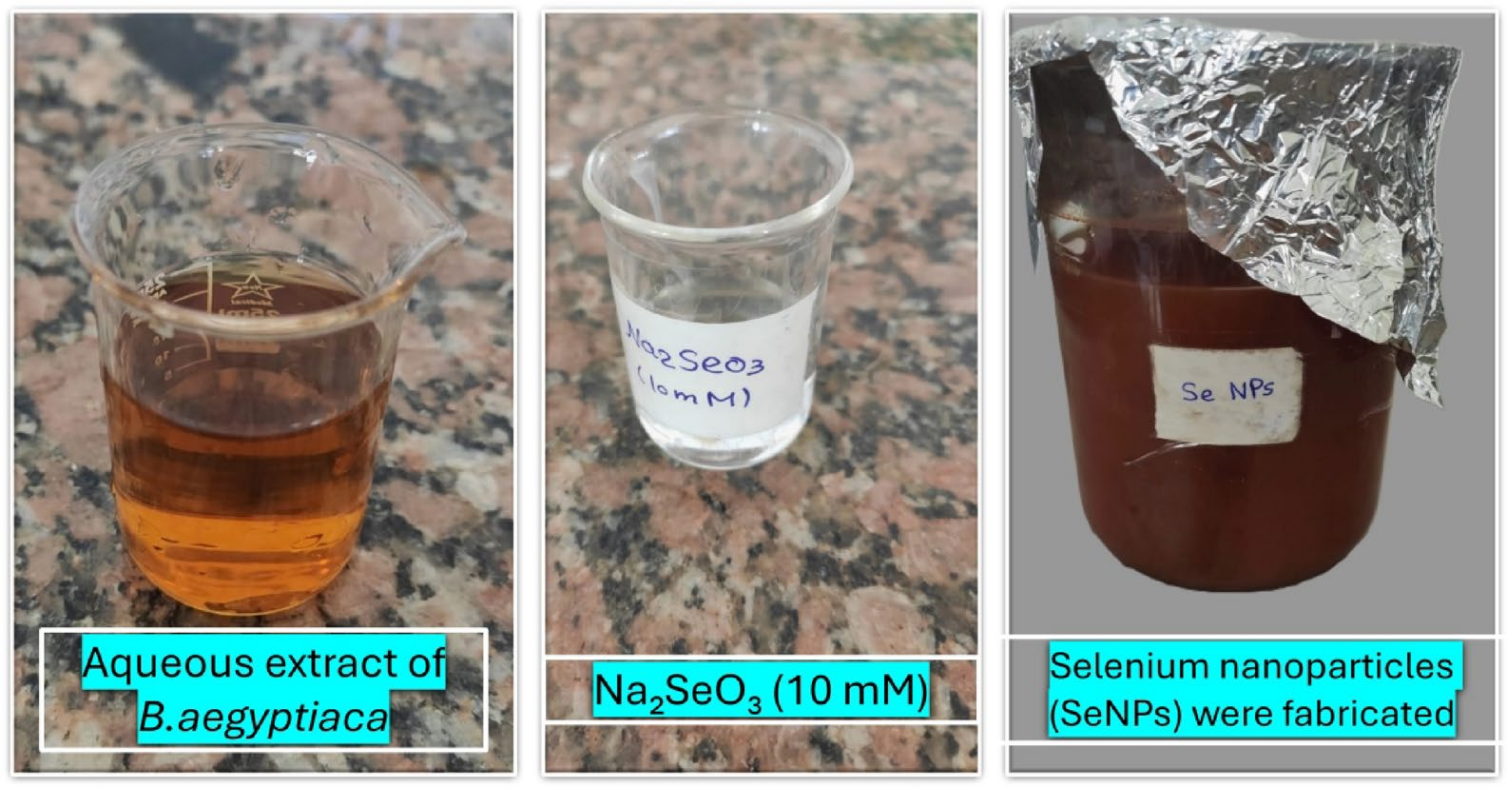
Color change of the reaction mixture during the biosynthesis of selenium nanoparticles (SeNPs).

The first sign that SeNPs are forming is a change in the hue of the reaction mixture. The hue transitioned from pale yellow to brick red, indicating the creation of SeNPs as shown in (Fig. 6). Se NPs made from an aqueous garlic (*A. sativum*) clove extract showed a similar pattern of visible light absorption^53^.

### Reduction of pH in reaction

The reaction mixture’s pH dropped from 8.04 to 5.28 when *B. aegyptiaca* mesocarp methanol extract was added, showing that 0.01 M (Na_2_SeO_3_) decreased during the production of SeNPs. The role of the extract in reducing pH during the biosynthesis of SeNPs is consistent with this discovery^53^. A change from a pale yellow to a brick-red color was observed in the Na_2_SeO_3_ solution when the initial pH was increased. *B. aegyptiaca* mesocarp methanol extract dissociation condition for capping functional groups may be affected by the pH of the reaction solution. A higher pH facilitates the deprotonation of the functional groups that cap the molecules. Deprotonated functional groups can display a stronger negative charge. The SeNPs are made more stable by electrostatic repulsion when the negatively charged groups contact them^54^.

### U.V. Spectrophotometric analysis

**Fig. 7:**
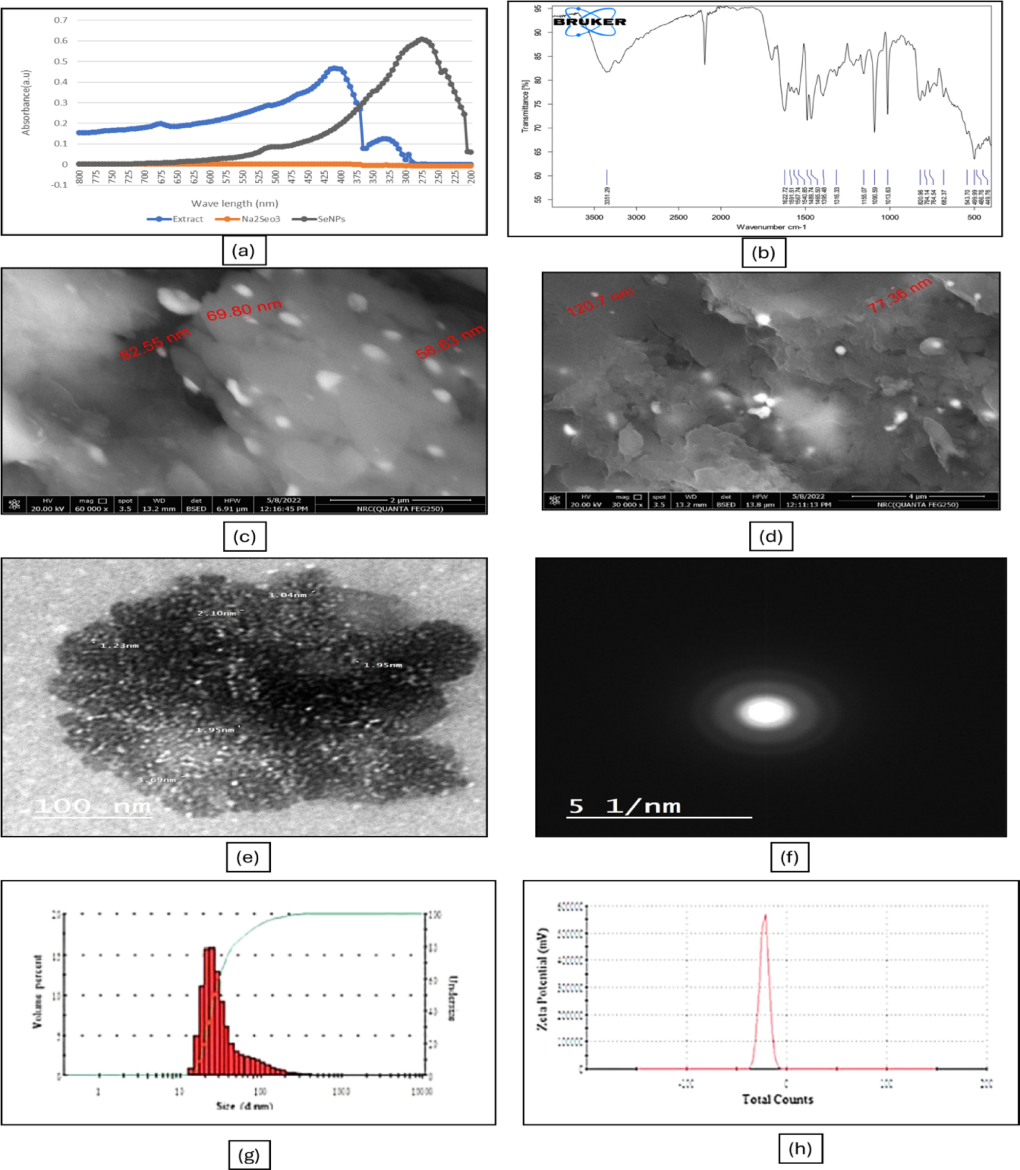
Schematic illustration of the characterization of green-synthesized selenium nanoparticles (SeNPs). (**a**) UV–Vis spectrophotometric analysis, (**b**) FTIR analysis, (**c**,** d**) FESEM images, (**e**) HR-TEM images, (**f**) SAED images, (**g**) Dynamic Light Scattering (DLS), and (**h**) Zeta potential of SeNPs.

The absorption spectrum of SeNPs produced by Green had a distinctive peak at 275 nm (Fig. 7a). The Surface resonance plasmon is the main factor that determines the optical absorption spectra of metal nanoparticles. This factor is influenced by particle size, shape, aggregation state, and the dielectric medium surrounding the particles^55,56^. There is a correlation between the SeNPs’ size and the features of the UV-visible spectra; when the particle size is 100 nm or larger, the visible spectrum displays clear, regular maxima, or kmax^57^. Approximately 25 nm is the size indicated by the spectra of the SeNPs. Observation of appropriate surface plasmon resonance (SPR) peaks near the 275 nm spectra shows that the methanol extract of *B. aegyptiaca* mesocarp effectively generated SeNPs. The stability and homogeneity of SeNPs are indicated by the peaks that consistently emerge around 275 nm over varied time intervals. At 265 nm, the SeNPs produced by the *Calendula officinalis* extract showed clear absorption bands^58^. Additionally, clove extract from garlic (*A. sativum*) exhibits clear absorption bands between 267 and 367 nm in the SeNPs absorption spectra^53^.

### FTIR analysis

FTIR measurements indicate the proportion of the transmission spectrum to determine whether a possible chemical reaction occurred between the extract and the nanoparticle. Additionally, functional groups have been defined. The FTIR spectra of *B. aegyptiaca*/ SeNPs are displayed in (Fig. 7-b). The FTIR spectra displayed a chemical reaction between the extract and the nanoparticles, showing distinctive bands due to several functional groups. The FTIR spectrum of *B. aegyptiaca*/ nanoparticles illustrated absorption bands at 3351.29 cm − 1 (O–H extending, phenols/alcohols), 2123.54 cm − 1 (C–H extending, alkyl), 1622.72 cm − 1 (C– –O extending), 1489.74 cm − 1 (skeleton motion of aromatic C– –C ring extending), 1316.33 cm − 1 (C–O extending, carboxyl acid),1155.07 cm − 1 (C–O extending, esters), 1090.59 cm − 1 (benzopyran ring motions), 1013.63 cm − 1 (C-H bending, alkenes) and 499.99 cm − 1 (C–I extending, halo compound).

### Field emission scanning electron microscopy (FESEM)

SeNPs produced by *B. aegyptiaca* mesocarp extract have a mostly spherical shape morphology, as seen in (Fig. 7-c, d) according to FESEM images. This result aligns with the spherical shape of the synthetic SeNPs produced from a water-based extract of the Horseshoe geranium plant^59^. Furthermore, SeNPs produced using *Catharanthus roseus* extract had a spherical shape^60^.

### HR-TEM analysis

The size and crystalline characteristics of the produced NPs can be examined via TEM analysis. The study was conducted using the JEOL-2100, and the images at different magnifications are shown in (Fig. 7-e). TEM pictures show the main series of SeNPs, which have an average size of approximately 1.95 to 3.69 nm. This result aligns with the synthesized SeNPs, which exhibited a spherical form and a size range of 10–80 nm when prepared using *Arauna* aqueous extract^61^. Furthermore, Mountain persimmon extract was used to create spherical SeNPs that had an average size of 4 to 16 nm^62^. Green SeNPs generated by B. aegyptiaca mesocarp methanol extract show a classic selected area electron diffraction (SAED) pattern with vivid circular rings symbolizing their extremely crystalline nature, as shown in (Fig. 7-f).

### Dynamic light scattering (DLS) and zeta potential analysis

The hydrodynamic diameter and size distribution of SeNPs were assessed using dynamic light scattering technique. The particles’ average size ranges from 10 to 500 nm, as shown in (Fig. 7-g). Furthermore, zeta potential analysis was used to determine the net charge on the surface of SeNPs and to understand their stability. The SeNPs zeta potential was found to be -35 mV (Fig. 7-h). This suggested an improvement in the NPs’ stability. High negative or positive (zeta potential) values in a suspension will cause the particles to reject one another and stop NP aggregation, according to Snehal Yedurkar et al.^63^. There isn’t any force stopping the particles from aggregating, though, if their zeta potential is low^64^.

### Cytotoxicity assay

**Fig. 8:**
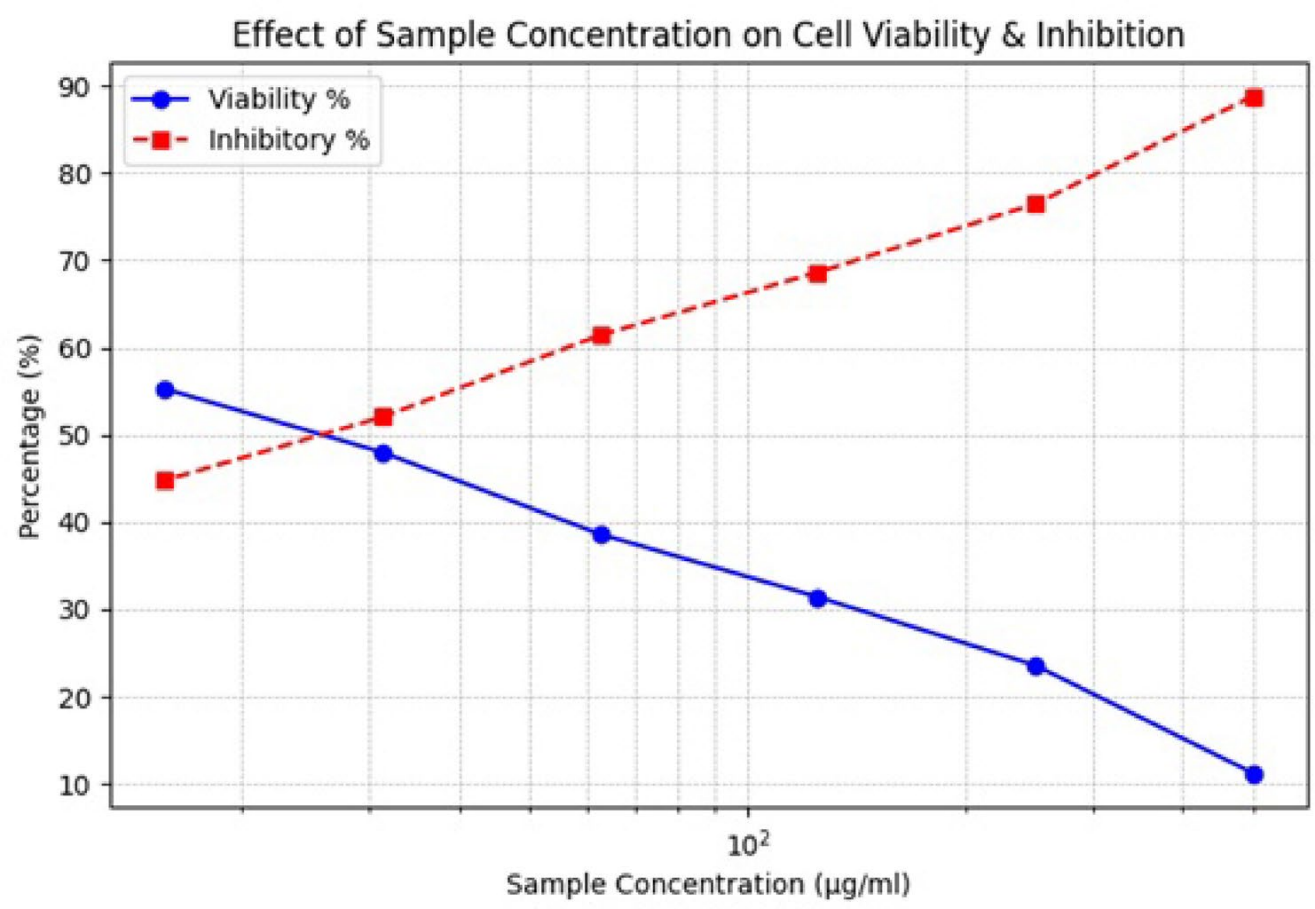
Line plot represents the percentage of cell viability and inhibition induced by SeNPs in cancer cell lines.

**Fig. 9:**
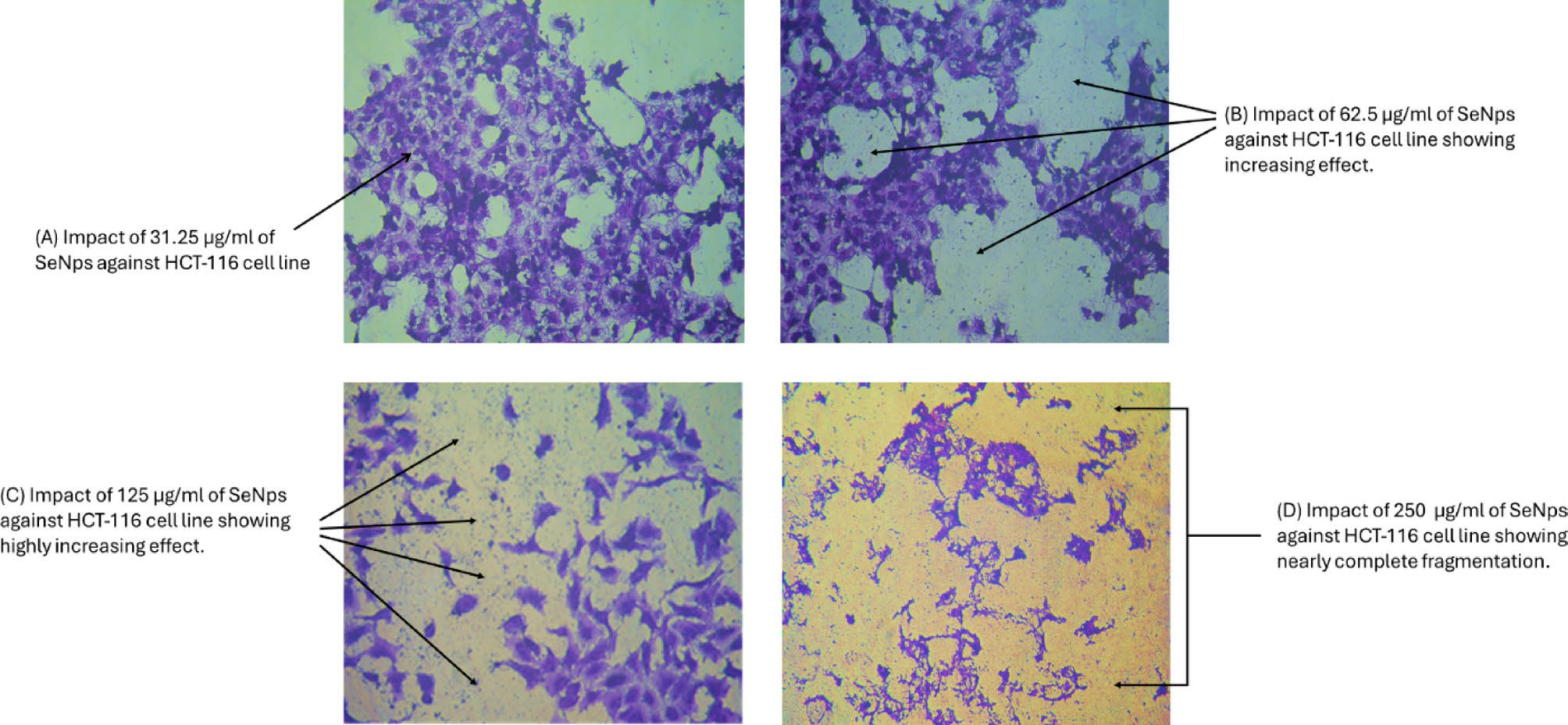
Anti-proliferative effect of selenium nanoparticles (SeNPs) on HCT-116 cell line. Images were captured using an inverted microscope at 200× magnification.

Due to their ability to enhance drug transport, increase bioavailability, and target cancer cells specifically while reducing systemic toxicity, nanoparticles (NPs) have significantly transformed cancer therapy^65^. Renowned for their unique properties and possible use in cancer treatment, SeNPs stand out among nanomaterials. These particles are ideal for targeting solid tumors, such as colorectal carcinoma, because their nanoscale size enables them to infiltrate tumors more effectively through the enhanced permeability and retention (EPR) effect^66,67^. Consistent with previous research, the current study suggests that SeNPs can preferentially target cancer cells, making them a promising candidate for the treatment of colorectal cancer. Their IC50 value of 30.03 demonstrated substantial potency in invading cancer cell lines when tested on commercial HCT-116 cells. Table 2; Figs. 8, and 9 show that different doses of selenium produce noticeable effects on cell viability and inhibition percentage. By inducing cell death in the HCT-116 cancer cell line through various pathways, SeNPs have demonstrated strong anticancer activity. Cancer cells experience oxidative stress due to the increased formation of reactive oxygen species (ROS) by cells exposed to SeNPs. By triggering caspase pathways and inducing mitochondrial malfunction, this imbalance leads to programmed cell death. It is possible to tackle the problem of drug resistance prevalent in traditional treatments by utilizing the fact that HCT-116 cells are more sensitive to SeNPs^68^. By increasing ROS production, SeNPs render cancer cells more susceptible to oxidative stress, which damages DNA and leads to cell death. On the other hand, healthy cells are protected from harm by the antioxidant properties of SeNPs^69^. Treatment of HCT-116 cells with selenium nanoparticles (SeNPs) resulted in pronounced morphological changes, as shown in Fig. 9. Compared to untreated control cells, the treated cells exhibited shrinkage, rounding, cytoplasmic condensation, and detachment from the culture surface. Additionally, a concentration-dependent decrease in cell density was observed, with higher concentrations of SeNPs leading to a greater reduction in the number of viable cells. These alterations indicate cellular stress and suggest the induction of apoptosis. Similar morphological and dose-dependent effects have been reported in previous studies on colon cancer cells treated with selenium nanoparticles^70–74^. Overall, these observations support the anti-proliferative potential of green-synthesized SeNPs.

### Molecular Docking

Molecular docking studies were carried out to better understand the binding energies (Kcal/mol) and amino acid residue interactions of (8) bioactive phenolic compounds isolated from *B. aegyptiaca* mesocarp methanol extract. The molecular docking results were compared to the co-crystalized ligand (GOL) entering into the binding site inside the active site of a CDK4 (PDB: 2W96) retrieved from the protein data bank (https://www.rcsb.org/structure/2W96) (accessed on 5 February 2022) to explore the binding mode.

**Table 2:** Anticancer effects of senps on HCT-116 colorectal cancer cells”.

Sample conc. (µg/ml)	Viability %	Inhibitory %	IC50
500	11.18	88.82	30.03
250	23.54	76.46	
125	31.38	68.62	
62.5	38.59	61.41	
31.25	47.97	52.03	
15.6	55.26	44.74	

**Fig. 10:**
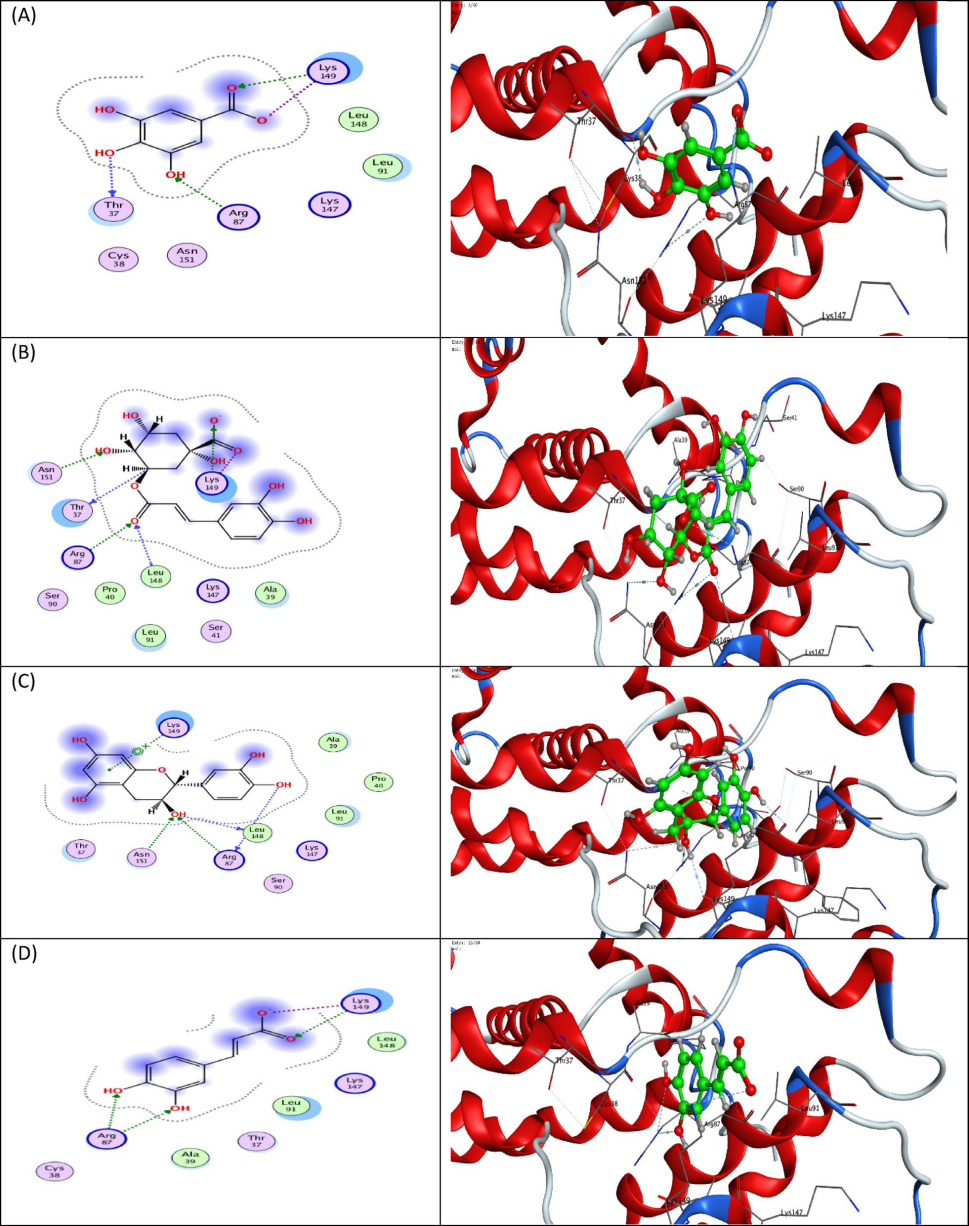

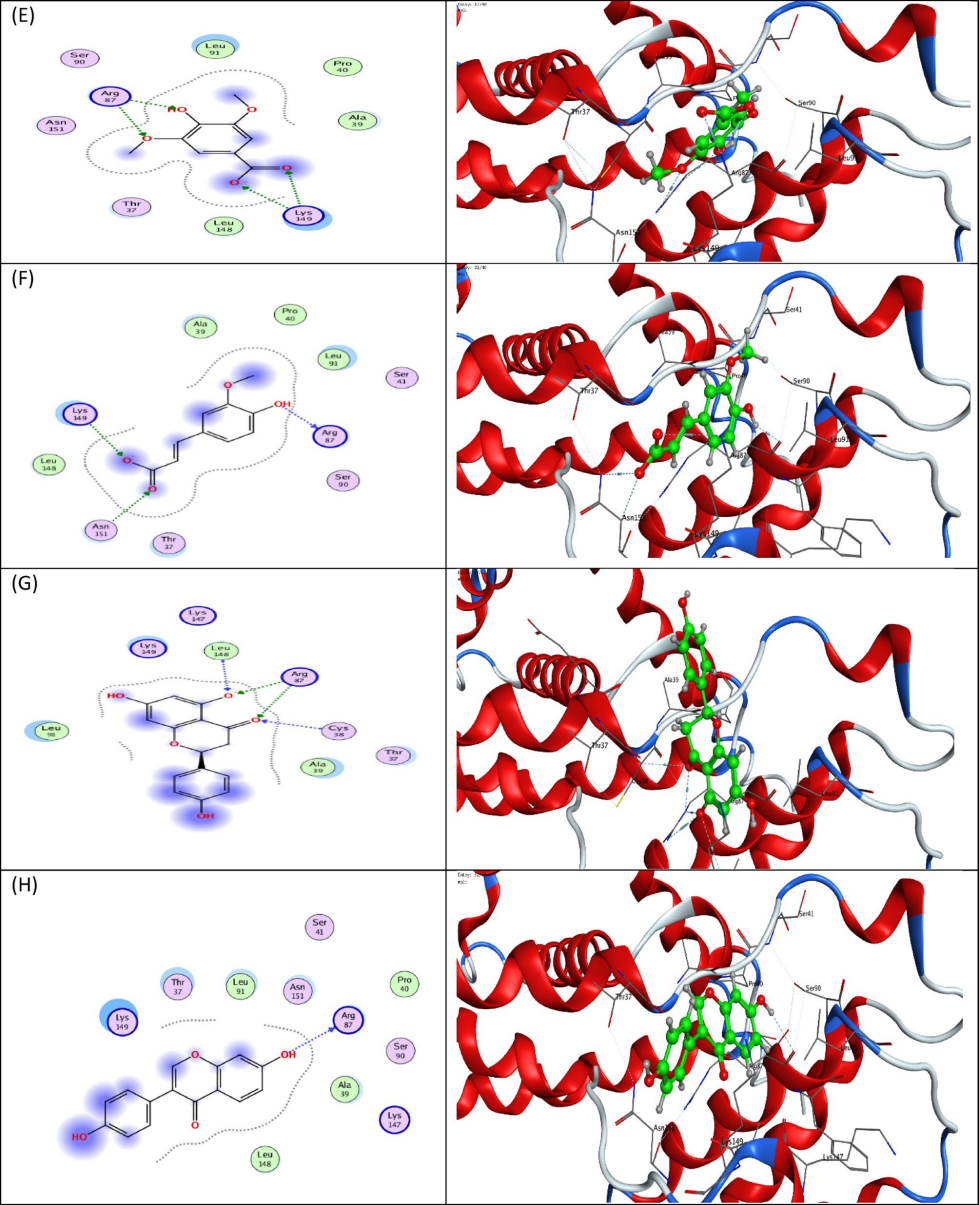
The 2D and protein–ligand interaction of each compound at the CDK4 enzyme active site (PBD ID: 2W96); (**A**) Gallic acid, (**B**) Chlorogenic acid, (**C**) Catechin, (**D**) Caffeic acid, (**E**) Syringic acid, (**F**) Ferulic acid, (**G**) Naringenin and (**H**) Daidzein.

According to the molecular docking analysis of compounds isolated from *B. aegyptiaca* mesocarp methanol extract with CDK4 protein. This study showed that 8 bioactive compounds from *B. aegyptiaca* mesocarp methanol extract were inferred to form interactions with good and acceptable binding affinity. The molecular docking analysis suggests a possible mechanism of action for these compounds. It showed that the four bioactive phenolic compounds catechin, naringenin, syringic acid and caffeic acid are the most potential compounds as CDK4 protein inhibitor as its strongest affinity and interaction in active site compared to CDK4 protein native ligands Glycerol (GOL) revealed binding free energy with a minus score, with free energies of -5.0051, -4.8779, -4.5103 and − 4.0738 kcal/mol, respectively as shown in (Fig. 10 & Table 3). Therefore, *B. aegyptiaca* mesocarp methanol extract is potentially an anticancer candidate, especially when carried on the surface of SeNPs, which may play a promising role in cancer-related diseases by regulating CDK4 protein activity.

**Table 3:** Molecular Docking study results of the bioactive phenolic compounds in *B. aegyptiaca* mesocarp methanol extract and the co-crystalized ligand (GOL), with binding energy and interacting groups with specific amino acids in residue inside the active site of the (CDK4) protein (PDB: 2W96) using MOE software*.

Compounds	Docking score (Kcal/mol)	RMSD	Ligand-receptor interactions
Co-crystalized ligand (GOL)	-3.4701	0.6332	2Arg87
Gallic acid	-3.8373	1.0297	Arg87, 2Lys149 and Thr37
Chlorogenic acid	-5.2145	1.2310	Arg87, Asn151, 2Lys 149, Leu148 and Thr37
Catechin	-5.0051	1.3854	2Arg87, Lys 149, Leu148 and Asn151
Caffeic acid	-4.0738	0.5767	2Arg87 and 2Lys 149
Syringic acid	-4.5103	0.8484	2Arg87 and 2Lys 149
Ferulic acid	-4.6602	0.9685	Arg87, Lys 149 and Asn151
Naringenin	-4.8779	1.4148	2Arg87, Leu148 and Cys38
Daidzein	-4.9681	0.9894	Arg87

### Antibacterial activity of *B. aegypticaca* and senps nanoparticles

**Fig. 11:**
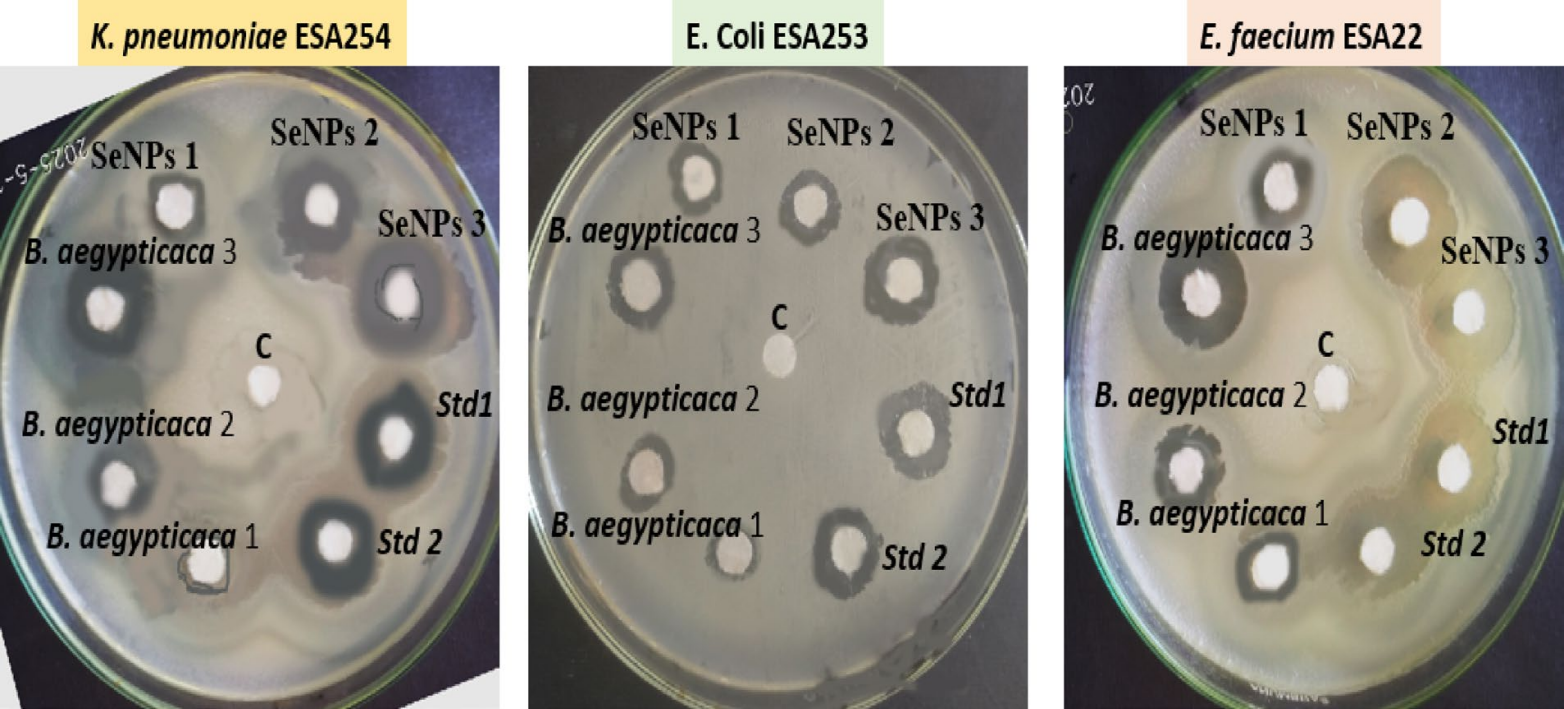
Showed the antibacterial activities of different concentrations of *B. aegypticaca* (50 µg/mL (*B. aegypticaca* 1), 100 µg/mL (*B. aegypticaca* 2), and 150 µg/mL (*B. aegypticaca* 3)], and different concentrations of SeNPs nanoparticles [50 µg/mL (SeNPs 1), 100 µg/mL (SeNPs 2), and 150 µg/mL (SeNPs 3)] against *K. pneumoniae* ESA254, and *E. coli* ESA253 pathogenes, and *Enterococcus faecium* ESA22 strains, compared with two antibacterial standards [10 µg/ml Gentamycin, (Std1) and 10 µg/ml Augmentin (Std2)] via a filter paper diffusion assay.

Inhibition zones demonstrated that the antibacterial assessment of *B. aegyptiaca* extract and biosynthesized SeNPs exhibited a clear dose-dependent effect. At 50, 100, and 150 µg/mL, the inhibition zones of SeNPs were consistently greater than those of the pure plant extract. The effectiveness of the plant extract is shown in Fig. 11. However, at a concentration of 150 µg/mL, SeNPs showed inhibitory zones measuring 17–18.5 mm against *K. pneumoniae*, *E. coli*, and *E. faecium*, surpassing these values. Based on the data in Table 4, it appears that SeNPs produced using environmentally friendly methods enhance the antibacterial efficacy of the bioactive chemicals discovered in *B. aegyptiaca.*

### Evaluating conventional antibiotics

The SeNPs exhibited comparable activity at higher concentrations to conventional antibiotics, including Augmentin and gentamicin. At 150 µg/mL, the inhibition zones of SeNPs were approximately 14–18 mm, which is similar to the range for gentamicin and amoxicillin (Augmentin). This suggests that SeNPs have the potential to function as efficient antimicrobial agents against both Gram-negative (*K. pneumoniae* and *E. coli*) and Gram-positive (*E. faecium*) bacteria, as demonstrated in Table 5.

### Minimum inhibitory concentration (MIC)

Additional evidence of SeNPs’ enhanced antibacterial activity compared to the crude plant extract was provided by the MIC values. Compared to the plant extract, which inhibited bacterial growth at concentrations of 68–128 µg/mL, SeNPs were able to do so at concentrations as low as 18–59 µg/mL. This indicates that nanoparticle formulation significantly enhances bioavailability and interaction with bacterial cells. With a minimum inhibitory concentration (MIC) of 18 µg/mL for SeNPs, *E. faecium* showed the most susceptibility among the pathogens evaluated, whereas *E. coli* showed the highest MIC at 59 µg/mL. The results show that SeNPs made using environmentally friendly methods increase the antibacterial efficacy of phenolic compounds from *B. aegyptiaca* and show promising action comparable to that of conventional antibiotics. Inhibition zones expanded in a dose-dependent manner and MIC values decreased, suggesting that SeNPs may be useful as a replacement or adjunctive antimicrobial agent in the battle against MDRs.

This confirms previous studies showing that *B. aegyptiaca* has strong antibacterial properties against a variety of pathogens, including *K. pneumoniae* and *E. coli*^75^. Similarly, SeNPs have been recognized for their strong antimicrobial capabilities, particularly against Gram-negative bacteria such as *K. pneumoniae* and *E. coli*^76^. The enhanced activity observed with SeNPs is likely associated with their unique physicochemical properties that facilitate better contact with the cell membranes of bacteria. This study opens the door to investigating other antimicrobial agents by demonstrating that *B. aegyptiaca* extract and SeNPs possess strong antibacterial properties.

**Table 4:** Antimicrobial activity of different concentrations (50, 100 and 150 µg/mL) of *B. aegypticaca* and senps nanoparticles against utis isolates; *K. pneumoniae* ESA254, and *E. coli* ESA253 pathogenes, and *E. faecium* ESA22 strains, compared with 10 µg/ml of Gentamycin and augmentin antibacterial standards.

Concentrations (µg/mL)		Inhibition Zone (mm)		
		K. pneumoniae ESA254	E. coli ESA253	E. faecium ESA252
*B. aegypticaca*	50	9.2 9.5 9.0	8.5 8.3 8.5	11.0 11.2 11.0
	100	12.5 13.0 12.5	11.5 10.9 11.5	12.7 12.5 13.0
	150	15.2 16.0 16.0	1.25 1.3 1.24	16.0 15.8 16.2
SeNPs	50	11.0 11.5 11.0	11.0 10.8 11.0	11.5 11.7 11.5
	100	15.3 15.0 15.5	12.5 12.0 12.8	16.0 16.5 16.0
	150	17.0 17.5 17.4	14.5 14.0 14.2	18.5 18.2 18.5
Gentamycin (Std1)	10	14.5 14.5 14.0	14.3 14.5 14.5	17.5 17.4 17.3
Augmentin (Std2)	10	15.2 15.5 15.0	12.5 12.8 12.5	18.0 18.2 18.0

**Table 5:** Minimum inhibitory concentration (MIC, µg/mL) of *B. aegypticaca* and senps nanoparticles against utis isolates; *K. pneumoniae* ESA254, and *E. coli* ESA253 pathogenes, and *E. faecium* ESA22 strains, compared with 10 µg/ml of Gentamycin and augmentin antibacterial standards.

Treatment	K. pneumoniae ESA254	E. coli ESA253	E. faecium ESA252
*B. aegyptiacaca* Extract	98	128	68
SeNPs	34	59	18
Gentamicin (Std1)	4	5	10
Augmentin (Std2)	2	3	6

### Cytological studies

The harmful effects of chemical fungicides on diseased plants can be discerned through cytological tests. According to an alternative explanation, cytological research constitutes biological control since it includes “the development of transgenic plants and biologically induced systemic resistance in hosts. The impact of various biological control agents on the *Vicia faba* plant, a key strategic crop. The negative effects of employing biological materials, such as nanoparticles and plant extracts, can be strongly demonstrated by cytological tests^77,78^.

Table 6 shows the effects of different SeNPs concentrations on mitotic indices (MI%), phase indices (PI%), cell types, and total abnormalities (Tab%). Most treatments showed a notable rise in the mitotic index following 24 h of exposure. The table shows that, compared to the control group’s 10.76% MI%, the lowest value was 9.11% at SeNPs 150, while the highest value was 13.87% at BaNS 50.

Increased cell division, as indicated by a higher mitotic index compared to the control, may have negative effects on the cell. Mitotic index readings were slightly lower when this therapy was applied. These results are in line with those reported by Liu et al.^79^ and may be a consequence of a longer G1 phase and an extended interphase, both of which hinder DNA synthesis and contribute to the decrease. Reductions or increases in the mitotic index have been shown to indicate the cytotoxicity levels of a chemical and can be used as a pollution monitor in the environment.

Compared to the control group, the results showed that the proportions of prophase, metaphase, anaphase, and telophase stages varied. This may be because different therapies are applied for varying durations during mitosis, which could result in different outcomes.

Values at the prophase stage showed both increases and decreases compared to the control group, indicating differences. When 50 SeNPs were present, the occurrence of prophase reached its peak at 19.65%. In comparison to the control group (20.65%), the treatment group showed the lowest frequency of prophase at 10.76%. Metaphase MI percentages increased; treatment BaNS 150 showed the greatest rise at 37.12%, while treatment 50 SeNPs showed the lowest meaningful increase at 31.87%. Without treatment, SeNPs 150, the percentage of MI in anaphase increased significantly (37.12%) compared to the control (30.14%), whereas all other treatments showed a small decline compared to the control. The percentage decreased compared to the control group showed a notable difference in the values of seeds treated with SeNPs during the anaphase stage. After 24 h of exposure, the highest frequency recorded was 33.54% at SeNPs 150, while the lowest was 22.32% at SeNPs 150, in comparison to the control.

When comparing the telophase stage values, the control value was 25.89%, while the treatment SeNPs 100 had the highest value at 25.03% and the lowest value at 18.58%. Compared to prophase, values increased during metaphase, anaphase, and telophase. At certain points in time, division has ceased, and the observed effects may be the consequence of treatments acting on the spindle. This confirms what Badr^29^, Selim^30^, and others have found, as well as what chitosan from squid pens does to *V. faba*^80^.

**Plate 1:**
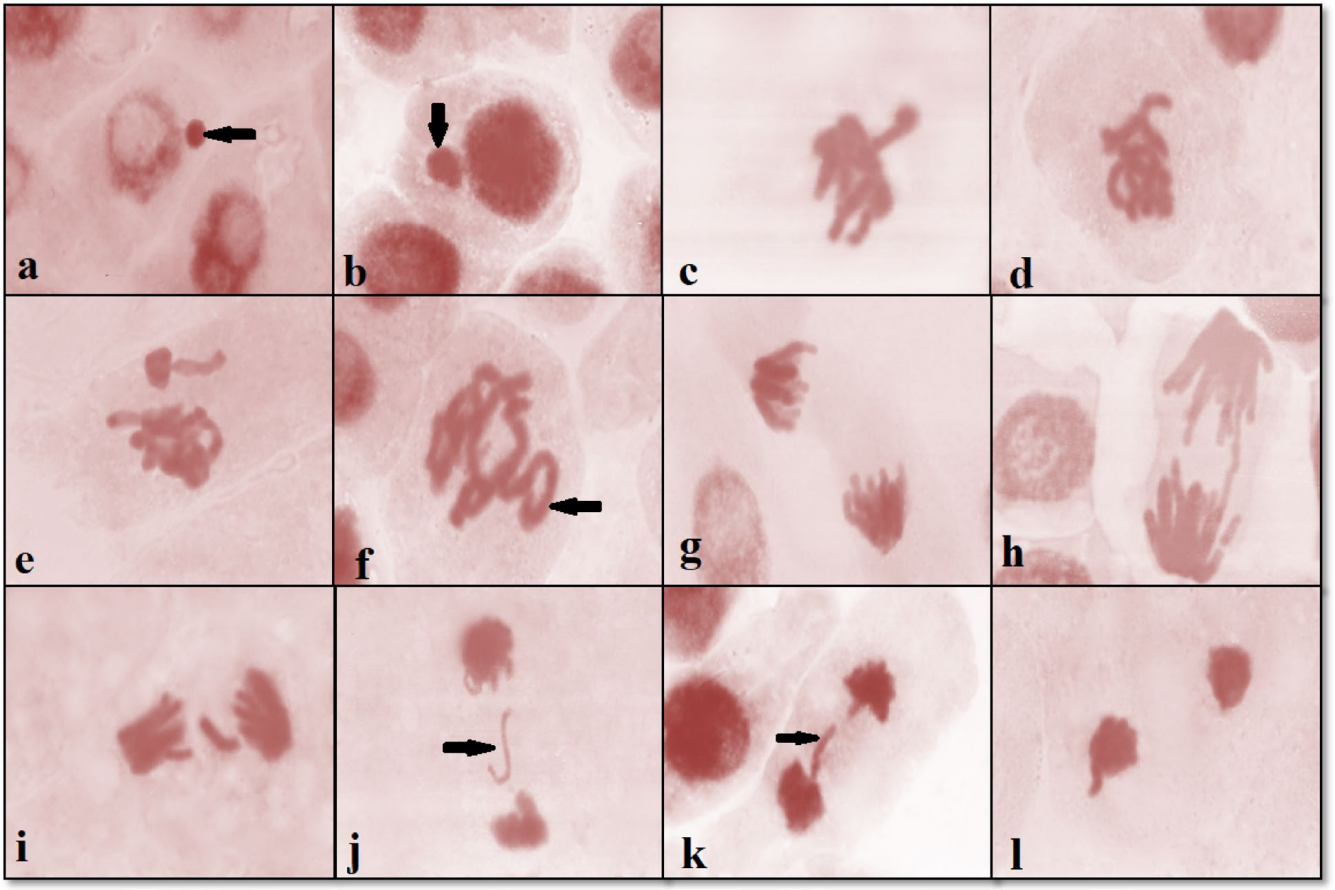
Chromosomal aberrations of *V. faba* root tips treated with different concentrations of SeNP and SeNPs: a&b: micronucleus at interphase, c: disturbed at metaphase, d: Stickiness at metaphase, e: non-congression at metaphase, f: ring at metaphase, g: diagonal at anaphase, h: bridge at anaphase, i: laggard at anaphase, J: laggard at telophase, k: late separation at telophase, l: disturbed.

Chemicals are considered genotoxic when they induce chromosomal abnormalities. During the interphase stage, this research detected anomalies, namely micronucleus cells. Plate 1 illustrates examples of stickiness, non-congression, rings, two groups, stars, and disturbances in metaphase, anaphase, and telophase, as well as late separation, diagonal, bridge, laggard, and anaphase disturbances.

Three types of chromosomal anomalies have been identified: Mitotic anomalies, which include c-metaphase, polyploidy, lagging, and multipolar division, make up the first category. The substances that alter the spindle apparatus are the cause of these disorders. The second category includes Cytomel breaks, sticky situations, and bridges. Micronuclei and multinucleated cells make up the third category.

Total abnormalities were lowest across all treatments (31.18% vs. 31.18% control). The total abnormalities percentage ranged from 8.29% at SeNPs 50 to 8.33% at SeNPs 100, with SeNPs 150 showing the highest rate at 20.49%.

As a potential replacement for chemical fungicides, which are both expensive and harmful, bio-agents like SeNP and 8Ba SNP are recommended. Overall concentrations of SeNPs resulted in significantly reduced aberrant mitosis compared to the control (dH_2_O).

**Table 6:** Mitotic indices, normal and abnormal phase indices, and total abnormalities in non-dividing and dividing cells after treating *Vicia Faba* root tips with different concentrations of *B. aegyptiaca* SeNPs.

Treatment		%MI	Phase index (PI)								% Total abnormal (Tab)	
			% Prophase		% Metaphase		% Anaphase		% Telophase		Interphase	Mitosis
Conc.	ET		Mitotic	Abn.	Mitotic	Abn.	Mitotic	Abn.	Mitotic	Abn.		
Control(dH_2_O)	**24**	10.76 ± 1.02	20.65	7.8	30.14	9.16	26.12	6.23	23.09	7.99	1.55 ± 0.02	31.18 ± 1.43
*B. aegyptiaca 50*	**24**	13.87 ± 0.97*	19.65	0.00	31.87	4.14	24.54	2.12	23.94	2.03	0.00 ± 0.00	8.29 ± 1.01ns
*B. aegyptiaca 100*	**24**	13.65 ± 0.91*	17.34	0.00	35.76	3.89	22.76	2.11	24.14	2.33	0.00 ± 0.00	8.33 ± 1.17ns
*B. aegyptiaca 150*	**24**	9.11 ± 0.34*	16.88	1.34	36.23	5.12	22.32	3.54	24.57	3.67	0.98 ± 0.01*	13.67 ± 1.32ns
*SeNPs 50*	**24**	15.77 ± 0.65*	17.12	0.32	32.54	5.22	24.65	4.11	25.69	5.11	0.00 ± 0.00	14.76 ± 1.54ns
*SeNPs 100*	**24**	12.87 ± 0.54*	16.21	0.14	33.65	4.21	25.11	3.66	25.03	4.07	0.00 ± 0.00	12.08 ± 1.79ns
*SeNPs 150*	**24**	9.18 ± 0.43*	10.76	2.11	37.12	8.15	33.54	5.11	18.58	5.12	1.58 ± 0.21	20.49 ± 1.98 *

*: significant ns: non-significant.

The present study demonstrates the successful eco-friendly synthesis of selenium nanoparticles (SeNPs) using the methanolic extract of *B. aegyptiaca* mesocarp and highlights their promising biological and cytogenetic properties^81,82^. The phytochemical analysis confirmed the presence of several bioactive phenolic compounds, including gallic acid, chlorogenic acid, and daidzein, which likely acted as natural reducing and stabilizing agents during nanoparticle formation^83^. These phytochemicals are well known for their redox activity and ability to cap metal nanoparticles, which explain the stability and uniformity observed in the produced SeNPs^66^. The green synthesis route thus not only avoids the use of hazardous chemicals but also adds intrinsic biological value to the final nanostructures through the presence of plant derived biomolecules^84^.

The structural and morphological characterization confirmed the formation of spherical SeNPs with an average diameter of approximately 2.82 nm, as revealed by TEM and FESEM analyses. The narrow particle size distribution and smooth surface morphology indicate controlled nucleation and growth, which can be attributed to the reducing potential of the phenolic compounds present in the extract. The UV–Vis spectral peak characteristic of SeNPs further validated their successful synthesis and optical stability. These findings demonstrate that B. aegyptiaca extract provides an efficient biogenic environment for nanoparticle synthesis, combining reduction and stabilization within a single-step process, and this confirms in previous studies^85–88^.

The biological evaluation of the biosynthesized SeNPs revealed potent cytotoxic activity against HCT-116 colorectal carcinoma cells. The nanoparticles significantly inhibited cell proliferation, showing an IC₅₀ of 30.03 µg/mL. This inhibitory effect may be associated with the generation of reactive oxygen species (ROS), which induces oxidative stress, mitochondrial dysfunction, and subsequent apoptosis^89^. The marked cytotoxic response observed suggests that the phenolic-functionalized surface of SeNPs may enhance their interaction with cellular components, leading to disruption of the cancer cell cycle and activation of programmed cell death pathways^90^. These results highlight the potential of SeNPs as a selective and effective anticancer agent, aligning with the emerging evidence that selenium-based nanostructures possess enhanced therapeutic performance compared with their bulk counterparts.

The synthesized SeNPs also exhibited remarkable antimicrobial activity against *K. pneumoniae*, *E. coli*, and *E. faecalis*. The inhibition zones increased in a dose-dependent manner, with the nanoparticles outperforming the crude plant extract and approaching the activity of standard antibiotics. Enhanced antibacterial efficacy can be attributed to the ultra-small size and large surface area of SeNPs, which facilitate close interaction with bacterial cell membranes, leading to disruption of membrane integrity and interference with essential metabolic processes. The results strongly support the view that green-synthesized SeNPs possess dual therapeutic potential, both anticancer and antimicrobial, making them promising candidates for biomedical and pharmaceutical applications^82,88^.

The cytogenetic evaluation using *V. faba* root tips provided further insight into the biological impact of SeNPs. Treatment with different nanoparticle concentrations influenced the mitotic index and induced concentration-dependent chromosomal aberrations, including laggards, bridges, and fragments. These findings indicate that while SeNPs can exert genotoxic effects at higher concentrations, their impact is dose-dependent and can be controlled within safe biological limits^91–93^.

Finally, the molecular docking analysis supported experimental observations by demonstrating strong interactions between the identified phenolic compounds and the active site of the CDK4 protein. These interactions suggest a possible molecular mechanism underlying the observed cytotoxic effects, where phenolic ligands may contribute to CDK4 inhibition and subsequent cell cycle arrest^94^. The integration of experimental and computational analyses, therefore, provides a comprehensive understanding of how B. aegyptiaca derived SeNPs exert their biological activity at both cellular and molecular levels.

Overall, this study establishes a clear link between the phytochemical composition of *B. aegyptiaca*, the physicochemical features of the synthesized SeNPs, and their multifunctional biological properties. The results collectively support the potential use of biogenic selenium nanoparticles as sustainable and effective agents in cancer therapy and antimicrobial applications. Further optimization and in vivo assessments are warranted to validate their therapeutic efficacy and safety for future biomedical translation.

## Conclusion

This study demonstrates the presence of bioactive phenolic compounds in the methanol extract of Balanites aegyptiaca mesocarp, which plays a key role in the green synthesis of stable, spherical selenium nanoparticles (SeNPs). The synthesized SeNPs exhibited notable antibacterial activity against both Gram-positive and Gram-negative bacteria and induced cytotoxic effects, including apoptosis, in HCT-116 colorectal cancer cells. Molecular docking analysis suggested that the phenolic components have a binding affinity for the CDK4 active site, which may contribute to their anticancer activity. Cytological analysis of Vicia faba root tips indicated concentration-dependent effects of SeNPs on mitotic activity and specific chromosomal abnormalities.

Although these results are promising, further in vivo studies and detailed mechanistic investigations are needed to confirm the therapeutic potential of SeNPs. Overall, the findings support the potential of green-synthesized SeNPs from B. aegyptiaca as a candidate for biomedical applications, while acknowledging the limitations of the current in vitro study.

## Supplementary Information

Below is the link to the electronic supplementary material.

Supplementary Material 1

## Data Availability

All data supporting the findings of this study are available within the paper and its Supplementary Information.
